# Effects of High-Temperature Heat Treatment Modification by Impregnation on Physical and Mechanical Properties of Poplar

**DOI:** 10.3390/ma15207334

**Published:** 2022-10-20

**Authors:** Jixiao Xue, Wei Xu, Jichun Zhou, Weiguo Mao, Shuangshuang Wu

**Affiliations:** 1 Co-Innovation Center of Efficient Processing and Utilization of Forest Resources, Nanjing Forestry University, Nanjing 210037, China; 2 College of Furnishings and Industrial Design, Nanjing Forestry University, Nanjing 210037, China

**Keywords:** urea-formaldehyde resin impregnation, high-temperature heat treatment, physical properties, mechanical properties, nano-SiO_2_

## Abstract

To expand the application range of fast-growing poplar, a modification method of poplar impregnated with nano-SiO_2_ and urea-formaldehyde resin was proposed in this study. Taking the mass ratio of nano-SiO_2_ mass to the solid content of urea-formaldehyde resin impregnation solution (W), high-temperature (H), and high-temperature time (T) as influencing factors, the effects of impregnation high-temperature heat treatment modification on the physical and mechanical properties of fast-growing poplar were explored. At the same time, the weight loss rate, oven-dry density, dry shrinkage properties, swelling properties, modulus of rupture (MOR), and modulus of elasticity (MOE) of the modified poplar were measured. The research results show that both the weight loss rate and the coefficient of variation of the oven-dry density have a high correlation with the temperature; the high-temperature immersion heat treatment can reduce the dry shrinkage and swelling of poplar, improve the dimensional stability, MOR, and MOE. W is 0–1%, H is 160 °C, and T is 2–4 h. The impregnated heat-treated wood has good MOR and MOE. Therefore, the combination of nano-SiO_2_ and urea-formaldehyde resin impregnation and heat treatment to modify poplar can improve some physical and mechanical properties of fast-growing poplar, expand the use of poplar, increase its added value, and realize high-value utilization.

## 1. Introduction

Wood is a renewable polymer material, which is widely used in construction, furniture, and interior decoration due to its excellent properties such as high strength-to-weight ratio and environmental protection. However, wood also has some natural defects, especially fast-growing forest wood often has problems such as low density, low physical and mechanical strength, and poor dimensional stability [1], which limits its application field. Wood modification is the main means to enhance the properties of fast-growing wood. Common processes include chemical modification (acetylation, furfurylation, and impregnation modification), heat treatment modification, and surface densification [2,3]. Numerous research results have been achieved in terms of color, mechanical strength, dimensional stability, flame retardancy, corrosion resistance, and multi-functional improvement.

High-temperature heat treatment of wood is an environmentally friendly technology, which is to place wood in a high temperature (160–250 °C) environment for heat treatment, which can be carried out in different media, such as air, water vapor, and nitrogen [4]. The wood after heat treatment is called carbonized wood, and the durability and stability are improved, and the color will change [5,6]. At present, the research on heat treatment mainly focuses on the influence of dimensional stability, mechanical properties, and wood color of the wood. In terms of dimensional stability, Wang et al. [7] used vacuum heat treatment to treat oak wood, which reduced the drying shrinkage and swelling of the wood and improved the dimensional stability. Quality control measures can usually be used to evaluate the quality of heat-treated materials, and color tests to evaluate the durability of heat-treated materials. Srinivas et al. [8] found that the thermal degradation rate was determined by the weight loss data, and the color change of wood after heat treatment using FTIR was mainly related to the cell wall polymer and to degradation. In terms of mechanical properties modification: Zhang et al. [9] proposed a process for heat-treating poplar with paraffin wax. H is 185 °C, T is 3.5 h, and the compressive strength in the grain and cross-grain directions is significantly increased. Bekir et al. [10] analyzed the difference in mechanical properties between young and mature eucalyptus wood after heat treatment and measured the modulus of elasticity (MOE), flexural strength (MOR), compressive strength (CS), and impact bending (IB); mechanical properties are reduced, but the reduction is greater in young wood.

To achieve a better wood modification effect, many scholars have studied the combined modification of impregnation and heat treatment. In terms of enhancing physical properties: Cui et al. [11] used scanning electron microscopy (SEM), Fourier transforms infrared light (FTIR), and X-ray diffraction (XRD) to measure the impregnated heat-treated material, and observed that the functional group structure changed, phase the dimensional stability of the comparative heat-treated material increases. There are also some studies showing that the immersion heat treatment process can reduce the water absorption and surface wettability of the wood, thereby improving the dimensional stability of the wood [12]. Cao et al. [13] studied the effect of impregnation heat treatment on the surface properties of poplar, which improved the surface roughness and color of poplar. In terms of mechanical properties, the resin impregnation heat treatment process can improve nail holding force, flexural strength, and flexural modulus of elasticity [14]. In previous studies, combinatorial modification often used the impregnation of wood followed by heat treatment. However, there are also some studies [15] that first heat-treated wood and then impregnated it, and they all used medium-temperature heat-treated wood to improve the physical and mechanical properties. In addition, some studies are also devoted to the functional modification of wood, and the combination of dipping and heat treatment is used to enhance the antibacterial properties of wood [16], anticorrosion [17], and flame retardancy [18].

Nanomaterials are the most popular modified materials in recent years [19] and have been applied to many modifiers, such as paints [20,21], wood, etc. It has been confirmed that nano-SiO_2_ can improve the mechanical properties of wood [22,23] and dimensional stability [24], and some scholars have also used SiO_2_ to improve the hydrophobicity and thermal insulation of wood [25]. Inspired by these aspects, in this study, nano-SiO_2_ was added to the urea-formaldehyde resin to impregnate the fast-growing poplar, and the integrated technology of impregnation enhancement and high-temperature heat treatment was used for modification. Considering that the combined modification will increase the time and cost, it is necessary to improve the process flow and parameters. It is found that the drying and curing in the later stage of the dipping process and the heating and drying in the early stage of the heat treatment process overlap transition time. By analyzing the weight loss rate, oven-dry density, dry shrinkage properties, swelling properties, modulus of rupture, and modulus of elasticity, the effects of high-temperature immersion heat treatment on the physical and mechanical properties of poplar were studied. This study can provide a reference for the academic community for the combination of impregnation and heat treatment to modify wood.

## 2. Materials and Methods

### 2.1. Materials and Equipment

#### 2.1.1. Experimental Materials and Equipment

The tree species selected in this study is the Chinese artificial fast-growing poplar (*Populus tomentosa*) with a size of 300 mm × 75 mm × 75 mm. They were provided by Suqian Poplar Wood Industry Co., Ltd. (Suqian, China). Air-dry to a moisture content of 8–12%, sawn and disassembled into two specimens of 300 mm × 20 mm × 20 mm and 20 mm × 20 mm × 20 mm. Defective pieces of wood are removed and sanded to make the surface smooth and clean. The specimens were put into a drying kiln for high temperature and dry treatment at 103 °C, and the moisture content was controlled at 0–3%. The purpose was to remove the free water in the wood so that the impregnating agent could be quickly filled into the wood longitudinal fiber The low molecular weight water-soluble urea-formaldehyde resin solution has a solids content of about 49.1% and a relative molecular weight of 300–500. Hydrophilic nano-SiO_2_, molecular weight 60.08, 99.8% purity, particle size 7–40 nm, specific surface area 200 m^2^/g. The nano-SiO_2_ was purchased from Shanghai Macklin Biochemical Co., Ltd. (Shanghai, China). The main experimental equipment includes an electronic balance (JCS-W, Harbin Zhonghui Weighing Instrument Co., Ltd., Harbin, China), a high-speed mixer (DF-101S, Gongyi Yuhua Instrument Co., Ltd., Zhengzhou, China), a vacuum pressure impregnation treatment tank (FCF-25L, Nanjing Keer Instrument Equipment Co., Ltd., Nanjing, China), a high-temperature drying oven, (XN—TH100, Jiangsu Xingnan Drying Equipment Co., Ltd., Nanjing, China), a constant temperature, humidity drying oven (HB-010, Nanjing Defu Experimental Equipment Co., Ltd., Nanjing, China), etc.

#### 2.1.2. Preparation of Modified Urea-Formaldehyde Resin Impregnant

The water-soluble urea-formaldehyde resin solution was diluted with distilled water, and the solid content was measured to be about 21.5%. Dry the nano-SiO_2_ particles, weigh according to the mass ratio of nano-SiO_2_ to the solid content of the urea-formaldehyde resin solution, and calculate according to Formula (1), slowly pour the nano-SiO_2_ into the urea-formaldehyde resin solution, and put it into a mixer for full stirring. The mixing speed is controlled at 1000 rpm–3000 rpm, and the mixing time is controlled at 30–90 min at 20–25 °C. The nano-SiO_2_ is fully dispersed in the urea-formaldehyde resin suspension to ensure that no agglomeration, condensation, or precipitation occurs.

(1) $$W=\frac{M_{1}}{M_{0}\times S}\times 100\;\%\;\;$$

where *W* is the mass ratio (%); $M_{1}$ is the mass of nano-SiO_2_ (g); $M_{0}$ is the mass of the urea-formaldehyde resin solution after dilution (g); *S* is the solid content of the urea-formaldehyde resin solution after dilution (%).

### 2.2. Experimental Design Scheme

#### 2.2.1. Experimental Design

The orthogonal experiment method is adopted, including three factors and three levels. The variable factors are the mass ratio of nano-SiO_2_ to the solid content of urea-formaldehyde resin (W), the high-temperature heat treatment temperature (H), and the high-temperature heat treatment time (T), which are divided into three levels: W = (0,1,2)%, H = (160, 180, 200) °C, and T = (2, 4, 6) h. In the preliminary experiments in this paper, the W value was selected as 0.5%, 1.5%, 2.5%, and 3.5% for impregnation. The results show that when the W value is 2.5%, 3.5%, the nano-SiO_2_ disperses in the impregnant for a long time, and it is easy to form agglomeration and precipitation, so choose W as 0%, 1%, 2% three levels. The orthogonal experiment is shown in Table 1. The vacuum degree, vacuum time, pressure, pressurization time, preheating time, drying time, low-temperature cooling time, etc. are fixed values.

#### 2.2.2. Impregnation High-Temperature Integrated Treatment

**Figure 1:**
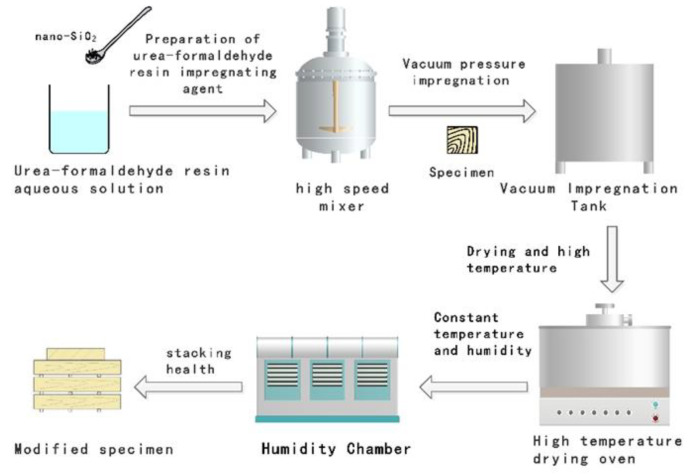
Impregnation high-temperature heat treatment integrated process.

The process flow of the high-temperature integrated treatment of impregnation is shown in Figure 1. First, the modified SiO_2_ and urea-formaldehyde resin impregnants are prepared, and the test pieces and impregnants are put into a vacuum impregnation tank for vacuum pressure treatment, left for a week after taking them out, and then put the specimens into a high-temperature drying box for medium-low temperature drying and high-temperature curing treatment. After heat treatment, they are placed in a constant temperature and humidity box for treatment. The treated specimens are stacked for health, and finally tested and stored.

Immersion high temperature integrated treatment steps:

1. Inject a fully stirred urea-formaldehyde resin-impregnating agent into the box, close the door of the impregnation tank after the wood is completely immersed in the liquid, and perform vacuum treatment. Then carry out pressure treatment, slow ly open the air valve to keep the internal and external air pressure consistent for 30 min; open the booster pump and the air valve of the immersion tank, and slowly increase the pressure to (0.7 ± 0.05) MPa, and maintain the pressure for 240 min. After the pressurization is completed, slowly release the pressure. After the internal and external pressures are balanced, open the tank door, take out the impregnating material, rinse the surface, and place it in a cool and ventilated place for 24 h.
2. Then put the impregnated material into the high-temperature treatment drying box for preheating, the speed is about 15 °C/h, the preheating temperature is 80 °C, and the preheating time is 6 h, and the actual time is adjusted according to the quantity of wood.
3. Rapidly increase the temperature to 103 °C, the speed is about 25 °C/h, and the constant temperature is 8 h, the moisture in the impregnating material is evaporated and the modified urea-formaldehyde resin is cured.
4. Slowly increase the temperature to 130 °C and keep the temperature constant for 2 h, further solidify the modified urea-formaldehyde resin inside the impregnating material, and also preheat slowly at high temperature.
5. Rapidly heat up to 160 °C, 180 °C, and 200 °C, keep the temperature constant for 2–6 h and spray steam (steam) appropriately during the treatment process.
6. Slowly cool down to 60 °C after heat treatment, the speed is about 10 °C/h, and the constant temperature is 3 h to prevent the wood from cooling too fast and causing warping deformation. The treated material is aged for health, the room temperature is 20–25 °C, the relative humidity is (65 ± 5)%, and it is left standing for more than 48 h to balance the moisture content.

### 2.3. Measurement and Characterization

#### 2.3.1. Statistical Analysis

This paper uses SPSS Statistics to conduct data statistics, range analysis, and variance analysis and uses Origin software to plot to analyze the impact of immersion heat treatment on the physical and mechanical properties of poplar. The Chauvenet criterion [26] is used to deal with unreasonable data in the experimental data. There are 15 specimens per group for each experiment on weight loss rate, oven-dry density, dry shrinkage properties, swelling properties, modulus of rupture, and modulus of elasticity.

#### 2.3.2. Weight Loss Rate (WLR)

The weight loss rate (WLR) is a value for judging the loss of wood after high-temperature treatment. The sample size is 20 mm × 20 mm × 20 mm. After preheating and drying at 103 °C, the immersed specimens were taken out and weighed immediately to record the absolute dry mass before treatment; after the heat treatment was completed, they were dried, and the samples were taken out and weighed immediately to record the absolute dry mass after heat treatment. The weight loss rate calculation Equation (2) is as follows:

(2) $$L=\frac{M_{I}{\;-\;M}_{H}}{M_{I}}\times 100\;\%\;$$

where *L* is the weight loss rate (%); $M_{I}$ is the absolute dry mass of the specimen before heat treatment (g); $M_{H}$ is the absolute dry mass of the specimen after heat treatment (g).

#### 2.3.3. Oven-Dry Density

Wood density can be divided into basic density, oven-dry density, and air-dry density. The oven-dry density is used in this paper, which is beneficial to exclude the factors of moisture in the wood, and can accurately reflect the amount of urea-formaldehyde resin and nano-SiO_2_ entering the interior of poplar and the heat treatment process loss of density. In this experiment, according to GB/T1933-2009 wood density determination method, the sample size is 20 mm × 20 mm × 20 mm. Calculated according to Equation (3), accurate to 0.001 g/cm^3^.

(3) $$\rho_{0}=\frac{M_{0}}{V_{0}}\;$$

where $\rho_{0}$ is the density of the sample when it is completely dry (g/cm^3^); $V_{0}$ is the volume f the sample when it is completely dry (cm^3^); $M_{0}$ is the mass of the sample when it is completely dry (g).

#### 2.3.4. Dry Shrinkage Property

The dimensional stability of wood mainly includes shrinkage and swelling. This article mainly discusses thread shrinkage, including full-drying shrinkage and air-drying shrinkage. When the moisture content is lower than the fiber saturation point, the wood will deform. Dry shrinkage is the amount of change in wood from dimensions above the fiber saturation point to dimensions in the dry state. Air-drying shrinkage refers to the change in the size of wood from a size above the fiber saturation point to when it reaches an equilibrium moisture content of 12%, determined according to GB/T1932-2009.

The oven-dry shrinkage Equation (4) in the radial and tangential directions is as follows, and the volumetric total dry shrinkage Equation (5) is as follows, accurate to 0.1%.

(4) $$\beta_{\max}=\frac{L_{\max}-L_{0}}{L_{\max}}\times 100\;\%\;$$

(5) $$\beta_{\mathrm{Vmax}}=\frac{V_{\max}-V_{0}}{V_{\max}}\times 100\;\%\;$$

where $\beta_{max}$ is the oven-dry shrinkage rate of the sample in the radial or chord direction (%); $L_{max}$ is the radial or tangential direction dimension of the sample when it is higher than the fiber saturation point (wet wood) (mm); $L_{0}$ is when the sample is completely dry radial or tangential dimension (mm); $\beta_{Vmax}$ is the total dry shrinkage of the sample volume (%); $V_{max}$ is the volume of the sample when it is wet (mm^3^); $V_{0}$ is the volume of the sample when it is completely dry (mm^3^).

The air-drying shrinkage rate Equation (6) in the radial and chord directions is as follows, and the volumetric total drying rate Equation (7) is as follows, accurate to 0.1%.

(6) $$\beta_{\omega }=\frac{L_{\max}-L_{\omega }}{L_{\max}}\times 100\;\%\;\;$$

(7) $$\beta_{V\omega }=\frac{V_{\max}-V_{\omega }}{V_{\max}}\times 100\;\%\;$$

where $\beta_{w}$ is the air-drying shrinkage of the sample in the radial or tangential direction (%); $L_{max}$ is the radial or tangential direction dimension of the sample when it is wet (mm); $L_{w}$ is the radial or tangential direction of the sample when it is air-dried dimensions (mm); *ω* is the moisture content of the sample when air-drying (%); $\beta_{V\omega }$ is the total dry shrinkage of the sample volume (%); $V_{max}$ is the volume of the sample when it is wet (mm^3^); $V_{\omega }$ is the volume of the sample when it is air-drying (mm^3^).

#### 2.3.5. Swelling Property

After wood absorbs water, its size and volume change with the moisture content. Swelling from full dryness to saturation refers to the ratio of the change in size or volume when the wood absorbs water to saturation to the size or volume when it is completely dry. The swelling property is measured according to the national standard GB/T1934.2-2009.

The radial or tangential linear swelling rate is calculated according to Equation (8), accurate to 0.1%; the volume swelling rate is calculated according to Equation (9), accurate to 0.1%.

(8) $$\alpha_{\max}=\frac{l_{\max}{-\;l}_{0}}{l_{0}}\times 100\;\%$$

(9) $$\alpha_{\mathrm{Vmax}}=\frac{V_{\max}{\;–\;V}_{0}}{V_{0}}\times 100\;\%$$

where $\alpha_{max}$ is the linear expansion properties of the specimen in the radial or chord direction (%); $l_{max}$ is the length of the specimen in the radial or chord direction (mm); $\alpha_{Vmax}$ is the volume expansion rate of the specimen (%); $l_{0}$ is the length of radial or tangential direction when the sample is completely dry (mm); $V_{max}$ is the volume when the sample absorbs water to be dimensionally stable (mm^3^); $V_{0}$ is the volume when the sample is completely dry (mm^3^).

#### 2.3.6. Modulus of Rupture (MOR) and Modulus of Elasticity (MOE)

The modulus of rupture (MOR) is a measure of the maximum fiber stress that wood resists bending. The modulus of elasticity (MOE) is the flexural deformation resistance of wood within a certain range, reflecting the degree of correlation between internal stress and strain. Both MOR and MOE were measured using 300 mm × 20 mm × 20 mm specimens, the length was along the grain direction, and a three-point bending test was carried out with a universal mechanical testing machine (AGS-X, Shimadzu, Japan).

The modulus of rupture (MOR) was determined according to GB/T1936.1-2009 national standard. The flexural strength of the sample when the moisture content is ω is calculated according to Equation (10), and the flexural strength when the moisture content of 12% is calculated according to Equation (11), accurate to 0.1 Mpa, and the moisture content is 9–15% range is valid by its formula.

(10) $$\sigma_{\omega }=\frac{{3P}_{\max}l}{{2\mathrm{bh}}^{2}}\;$$

(11) $$\sigma_{12}{=\sigma }_{\omega }\left[1+0.04\left(\omega \;–\;12\right)\right]$$

where $\sigma_{\omega }$ is the flexural strength of the sample when the moisture content is *ω* (Mpa); $P_{max}$ is the failure load (N); *l* is the span between the two supports (mm); *b* is the width of the sample (mm); *h* is the height of the sample (mm).

The modulus of elasticity (MOE) was determined according to GB/T1936.2-2009 national standard. When the moisture content of the sample is *ω*, the flexural modulus of elasticity is calculated according to Equation (12), accurate to 10 Mpa. The flexural strength when the moisture content is 12% is calculated according to Equation (13), accurate to 0.1 Mpa, and the formula is valid when the moisture content is in the range of 9–15%.

(12) $$E_{\omega }=\frac{{23\mathrm{Pl}}^{3}}{{108\mathrm{bh}}^{3}f}\;$$

(13) $$E_{12}{=E}_{\omega }\;[1+0.015(\omega -12)]\;\;$$

where $E_{\omega }$ is the flexural modulus of elasticity when the moisture content of the sample is *ω* (Mpa); *P* is the difference between the upper and lower limit loads (N); *l* is the span between the two supports (mm); *b* is the width of the sample (mm); *h* is the height of the specimen (mm); *f* is the deformation value of the specimen between the upper and lower limit loads (mm). $E_{12}$ is the flexural strength of the sample when the moisture content is 12% (Mpa); *ω* is the moisture content of the sample (%).

## 3. Results and Discussion

### 3.1. Weight Loss Rate and Absolute-Dry Density

#### 3.1.1. Weight Loss Rate Analysis

As shown in Table 2, the high temperature is the biggest factor affecting the weight loss rate, followed by high-temperature time, and the addition of nano-SiO_2_ has little effect on the weight loss rate. Among them, when W is 0%, H is 200 °C, and T is 6 h, the mass loss is the largest; when W is 2%, H is 160 °C, and T is 6 h, the mass loss is the smallest.

As shown in Table 3, the meaning of * is 0.05 < *p*-value < 0.1. The high temperature has a significant effect on the weight loss rate of the treated wood at the significance level of 0.1, and the W and time have little significance. That is, high temperature is the main factor affecting the weight loss rate.

#### 3.1.2. Absolute-Dry Density Analysis

The amount of density change can also be used to evaluate the effect of the heat treatment process on the specimen. Similar to the mass change, the full dry density also decreased compared with that before heat treatment, and the amount of change was related to W, H, and T. In this experiment, the density drop ranged from 0.005 g/cm^3^ to 0.028 g/cm^3^, the density variation coefficient was 0.9–4.7%, and the variation was small. It can be seen from Table 4 that when W is 0%, H is 200 °C, and T is 6 h, the coefficient of variation of oven-dry density is the largest; when W is 2%, H is 160 °C, and T is 6 h, the coefficient of variation of oven-dry density is the smallest. It can be seen from Table 5, the meaning of ** is 0.01 < *p*-value < 0.05, temperature is the biggest influencing factor of density change, with high significance. The effects of W and T are general and not significant.

### 3.2. Dry Shrinkage Property

#### 3.2.1. Visual Analysis of Oven-Dry Shrinkage and Air-Dry Shrinkage

Total dry shrinkage rate analysis: It can be seen from Table 6 that for the radial, chordwise, and volume oven-dry shrinkage rates, the temperature H is the largest, followed by the high-temperature time T, and the difference between the mass of nano-SiO_2_ and the solid content of the urea-formaldehyde resin solution The range of mass ratio W is the smallest, indicating that H is the most influential factor, followed by T, and nano-SiO_2_ has the smallest influence. Among them, to achieve the smallest radial dry shrinkage, the parameters can be adjusted to W is 0% or 1%, 200 °C high temperature, high temperature 6 h; when W is 0%, 200 °C high temperature, high temperature 6 h, the chord direction, and volume are full. Dry-to-dry shrinkage is minimized. To sum up, if the total dry shrinkage of the modified material is to be minimized, the parameters can be adjusted to W = 0%, high temperature of 200 °C, and high temperature of 6 h.

Analysis of air-drying shrinkage rate: As shown in Table 6, similar to the full-drying shrinkage rate, the range R is H, T, and W in descending order, indicating that the heat treatment temperature has the greatest influence on the air-drying shrinkage rate, and high temperature. The effect of time is smaller than that of temperature, and the addition of nano-SiO_2_ has the smallest effect on the air-drying shrinkage rate. At the same time, when W is 0%, H is 200 °C, and T is 6 h, the air-drying shrinkage rate of immersion heat-treated modified poplar reaches the minimum.

#### 3.2.2. Visual Analysis of Oven-Dry Shrinkage and Air-Dry Shrinkage

As shown in Table 7, the meaning of ** is 0.01 < *p*-value < 0.05, the effect of temperature and the high-temperature time on the radial dry shrinkage rate of poplar specimens is very significant at the level of 0.05, while the effect of nano-SiO_2_ on the radial dry shrinkage rate is not significant. According to Table 8 W, H, and T have no significant effect on the dry shrinkage of poplar tangential direction at the 0.1 level. In Table 9, the meaning of * is 0.05 < *p*-value < 0.1, the volume drying shrinkage rate is more significant at the significance level of 0.1, and the other factors are not obvious. In summary, the temperature is an important factor affecting the dry shrinkage rate, and the addition of nano-SiO_2_ has the least effect.

The meaning of * is 0.05 < *p*-value < 0.1, ** is 0.01 < *p*-value < 0.05. It can be seen from Table 10, Table 11 and Table 12 that the effect of temperature on the radial and volume air-drying shrinkage of poplar is significant at the level of 0.1. However, W, temperature, and high-temperature time were not significant for the air-drying shrinkage of poplar at the level of 0.1. In summary, the temperature is an important factor affecting the air-drying shrinkage rate of modified woods, and the high-temperature time and the addition of nano-SiO_2_ have less effect. By comparing the variance results of the two drying shrinkage ratios, the heat treatment temperature drying shrinkage ratio has an important influence and is significant. The effect of high-temperature time is relatively small, and the effect of nano-SiO_2_ is the smallest.

#### 3.2.3. Comprehensive Comparative Analysis of Drying Shrinkage

**Figure 2:**
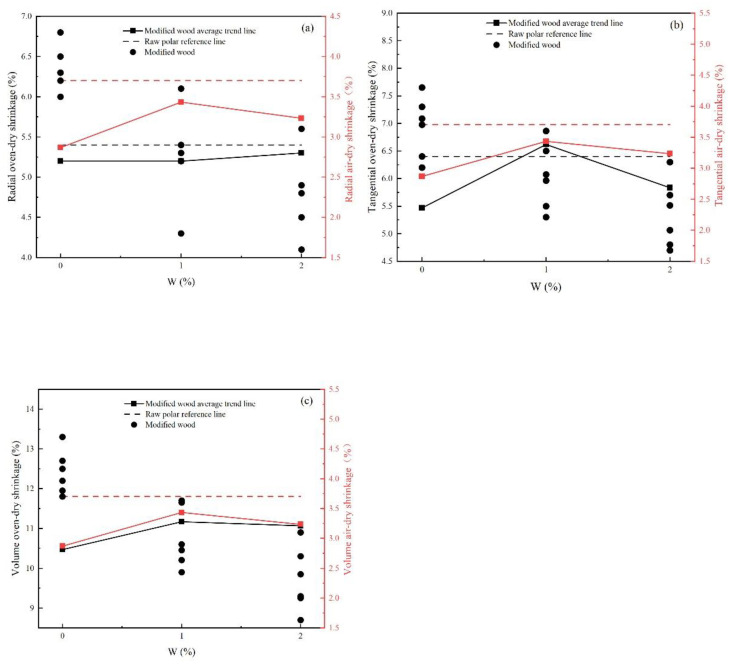
Comparative analysis of dry shrinkage of modified wood based on W: (**a**) radial oven-dry and air-dry shrinkage; (**b**) tangential oven-dry and air-dry shrinkage; (**c**) volume oven-dry and air-dry shrinkage.

The dry shrinkage rate of the modified wood presents an irregular distribution. When the W is the same, the dry shrinkage rate changes reflected by different temperatures and times are different. From Figure 2a–c, according to the trend line of the average dry shrinkage rate of the modified wood, the change and trend of the dry shrinkage rate of the modified wood can be vaguely evaluated. The radial shrinkage basically increases with W increasing; the tangential shrinkage rate basically showed a downward trend and was first urgent and then slow; The shrinkage rate in the volume direction is basically similar to that in the Tangential direction.

As shown in Figure 2, the dry shrinkage rate of the modified wood is basically lower than the comparison of the dry shrinkage rate of the material, indicating that the high-temperature modification has an obvious effect on the dimensional stability of poplar. Secondly, from the distribution point of view, the temperature above the reference line of the drying shrinkage rate of the material is basically 160 °C, that is, the effect of high temperature on the drying shrinkage rate should be higher than 160 °C. Shrinkage is similar.

### 3.3. Swelling Property

#### 3.3.1. Visual Analysis of Swelling Property

As shown in Table 13, from dry to water saturation, the radial swelling rate is 3.3% to 4.2%, the tangential direction is 3.9% to 5%, and the volume is 7.3% to 9.2%. The radial swelling rate is high temperature. The H range is the largest, H > W > T, it can be understood that the influence of H on the swelling property is greater than that of W and T; the chordwise swelling ratio range is W > H > T, that is, W has the greatest influence on the swelling property factor; the volume expansion rate is extremely poor H > W > T, and temperature is the most important factor. When W is 0%, H is 200 °C, T is 6 h, the radial swelling property, the tangential swelling property, and the volume swelling property are the minimum.

#### 3.3.2. Analysis of Variance of Swelling Property

As shown in Table 14, the meaning of * is 0.05 < *p*-value < 0.1, the effect of temperature is significant at the level of 0.1; according to Table 15, it can be seen that the effects of W, H, and T on the swelling properties are not significant; from Table 16, the meaning of * is 0.05 < *p*-value < 0.1, ** is 0.01 < *p*-value < 0.05., at the level of 0.1, the temperature effect is very significant, and nano-SiO_2_ impact is also significant. In summary, the temperature is the most important factor affecting the swelling property, and nano-SiO_2_ also plays an important role.

#### 3.3.3. Comparative Analysis of Swelling Property

**Figure 3:**
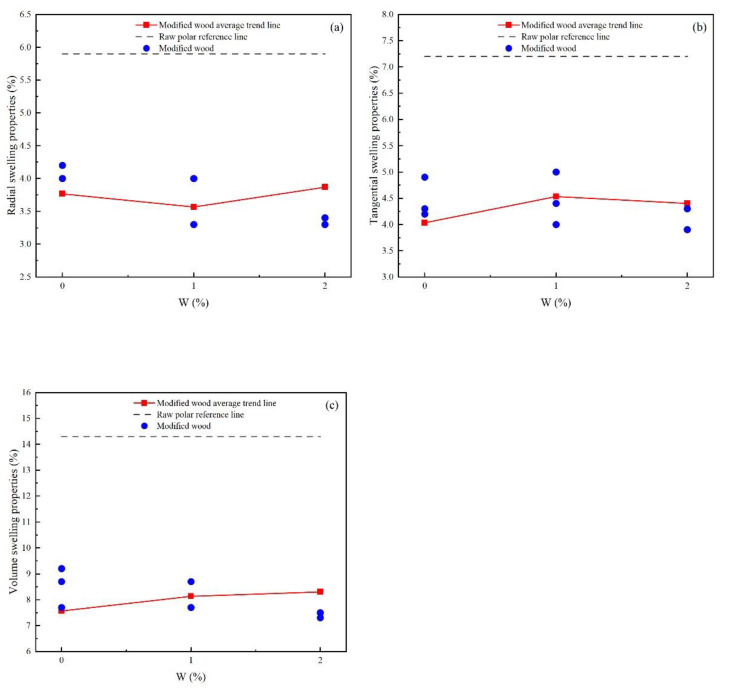
Comparative analysis of swelling properties of modified wood based on W: (**a**) radial swelling properties; (**b**) tangential swelling properties; (**c**) volume swelling properties.

As shown in Figure 3a–c, from the perspective of poplar material and modified material, the radial, Tangential, and volumetric swelling rates of modified materials are much smaller than those of the material, and the immersion high-temperature heat treatment modification can effectively reduce poplar wood. The swelling rate, improves the dimensional stability of wood.

With the increase of W, although the average swelling ratio of the modified material cannot fully represent the actual swelling ratio, the trend of the swelling ratio based on W can be judged. From the trend of the average swelling rate, with the increase of W, the swelling rate rises and falls, and the trend is not obvious.

### 3.4. Modulus of Rupture (MOR) and Modulus of Elasticity (MOE)

#### 3.4.1. Visual Analysis of MOR and MOE

From Table 17, it can be seen that high temperature has the greatest impact on the MOR and MOE of wood, and the addition amount of nano-SiO_2_ material and high-temperature time have a general effect on the bending resistance. When the mass ratio W of nano-SiO_2_ and urea-formaldehyde resin is dissolved and solid content is 1%, H is 160 °C, high temperature is 2 h, MOE reaches the optimum value, and MOR reaches the optimum value when T is 4 h.

#### 3.4.2. Analysis of Variance of MOR and MOE

Table 18 and Table 19 judges whether the influence of the three influencing factors on the bending resistance is significant. As shown in the Tables, the meaning of * is 0.05 < *p*-value < 0.1, at a Significant level of 0.1, the high temperature has a significant effect on the flexural strength and flexural modulus of elasticity, and the amount of nano-SiO_2_ has a limited effect on the high-temperature time.

#### 3.4.3. Comparative Analysis of MOR and MOE between Material and Modified Material

**Figure 4:**
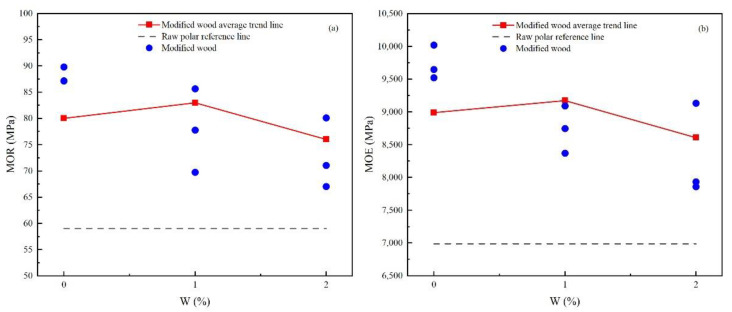
Comparative analysis of MOR and MOE of modified wood based on W: (**a**) MOR; (**b**) MOE.

As shown in Figure 4a, the flexural strength of the modified wood is greater than that of the raw polar, indicating that impregnation with modified urea-formaldehyde resin can effectively improve the flexural strength of the wood. The urea-formaldehyde resin penetrates the wood and plays a role in connecting and bonding the wood fibers of the wood, strengthening the bending ability and toughness of the fibers. The high temperature makes the urea-formaldehyde resin solidify and becomes brittle, and the wood fibers have a tendency to carbonize, thereby reducing the ability of the fibers to bend, that is, the wood “becomes brittle”. The addition of nano-SiO_2_ also affects flexural strength. With the increase in the amount of SiO_2_, the flexural strength tends to increase first and then decrease.

As shown in Figure 4b, the flexural elastic modulus of the modified wood is similar to the flexural strength. The flexural elastic modulus of the fast-growing poplar is about 7000 MPa, and the MOE of the modified poplar is higher than that of the modified woods, indicating that the urea-formaldehyde resin impregnation heat treatment modification can improve the flexural elastic modulus of wood to a certain extent. When the temperature is higher and the time is longer, the MOE is worse. The addition of nano-SiO_2_ also affects the MOE. With the increase of W, the MOE of the impregnated heat-treated wood increases first and then decreases.

## 4. Conclusions

In this study, fast-growing poplar was modified by impregnation and high-temperature heat treatment, and the effect of the modified formula on the physical and mechanical properties of poplar was analyzed. The main conclusions are as follows:

1. The temperature during heat treatment is the most influential factor on the weight loss rate, followed by the heat treatment time, and nano-SiO_2_ has little effect on the weight loss rate. Both the weight loss rate and the coefficient of change of full dry density have a high correlation with temperature. When W is 0%, H is 200 °C, and T is 6 h, the coefficient of change of weight loss rate and oven-dry density is the largest; when W is 2%, H is 160 °C, T is 6 h, the variation coefficient of the two is the smallest.
2. Impregnation high-temperature heat treatment can appropriately reduce the dry shrinkage properties and improve dimensional stability. When W is 0%, H is 200 °C, and T is 6 h, the dry shrinkage rate of poplar is the smallest, and the dimensional stability is in the best state.
3. The urea-formaldehyde resin impregnation high-temperature heat treatment modification can effectively reduce the swelling rate of poplar fast-growing wood and improve its dimensional stability. When W is 0%, H is 200 °C, and T is 6 h, the radial swelling property, the tangential swelling property, and the volume swelling property are optimal.
4. The modulus of rupture (MOR) and modulus of elasticity (MOE) of the modified wood are improved to a certain extent compared with the raw polar. When W is 0–1%, H is 160 °C, and T is 2–4 h, the impregnated heat-treated wood has good MOR and MOE. The flexural properties of fast-growing poplar are also affected by moisture content, density, and other factors. Within a certain range, the lower the moisture content, the higher the bending resistance of the modified poplar.

In this paper, nano-SiO_2_ is added to the urea-formaldehyde resin impregnating agent to impregnate poplar, and the integrated modification method is adopted, which simplifies the process flow, shortens the time and cost, and improves the performance of poplar. The method can expand the application field of fast-growing poplar and guide the preparation of high-value wood functional materials. The addition of nano-SiO_2_ can have a positive impact on the MOR and MOE of poplar to a certain extent, and the proper amount of SiO_2_ can improve the mechanical properties of poplar, but it has no obvious impact on the weight loss rate, total dry density and dimensional stability of poplar. However, the applicability of this method to the industrial field for large-scale samples needs to be explored and demonstrated in the future.

## Figures and Tables

**Table 1:** Orthogonal test of dipping high-temperature treatment.

Number	Factor		
	W (%)	H (°C)	T (h)
1	0	160	2
2	0	180	4
3	0	200	6
4	1	160	4
5	1	180	6
6	1	200	2
7	2	160	6
8	2	180	2
9	2	200	4

**Table 2:** Visual Analysis of weight loss rate of impregnated heat-treated wood.

Number	W (%)	H (°C)	T (h)	L (%)
1	0	160	2	1.853 (12.65)
2	0	180	4	3.269 (6.07)
3	0	200	6	6.238 (9.34)
4	1	160	4	1.893 (9.14)
5	1	180	6	3.635 (7.52)
6	1	200	2	3.694 (6.05)
7	2	160	6	1.797 (13.97)
8	2	180	2	2.468 (9.64)
9	2	200	4	5.353 (9.26)
Mean 1	3.787	1.848	2.672	
Mean 2	3.074	3.124	3.505	
Mean 3	3.206	5.095	3.890	
Range R	0.713	3.247	1.218	

**Table 3:** Variance analysis of weight loss rate of impregnated heat-treated wood.

Factor	Sum of Squares	Degree of Freedom	F Ratio	F Critical Value	*p*-Value	Significance
W	0.862	2	0.999	9	0.500	
H	16.059	2	18.608	9	0.051	*
T	2.327	2	2.696	9	0.271	
error	0.863	2				

Significant level α = 0.1.

**Table 4:** Analysis of total dry density range of impregnated heat-treated wood.

Number	W (%)	H (°C)	T (h)	$\rho_{1}$	$\rho_{H}$	$\Delta_{\rho_{1}-\rho_{H}}$	$K_{\rho_{1}-\rho_{H}}$
1	0	160	2	0.589 (4.8)	0.581 (4.8)	0.008	1.4
2	0	180	4	0.587 (4.3)	0.575 (4.3)	0.012	2.0
3	0	200	6	0.602 (6.3)	0.574 (6.9)	0.028	4.7
4	1	160	4	0.580 (7.3)	0.573 (7.3)	0.007	1.2
5	1	180	6	0.582 (7.8)	0.567 (8.0)	0.015	2.5
6	1	200	2	0.584 (5.3)	0.567 (5.4)	0.017	2.9
7	2	160	6	0.564 (5.6)	0.559 (5.6)	0.005	0.9
8	2	180	2	0.600 (8.1)	0.590 (8.4)	0.010	1.7
9	2	200	4	0.576 (7.6)	0.552 (7.6)	0.024	4.2
Mean 1	2.7	1.167	2	2.7			
Mean 2	2.2	2.067	2.467	2.2			
Mean 3	2.267	3.933	2.7	2.267			
Range R	0.5	2.766	0.7	0.5			

Where ”$\rho_{1}$” is the oven-dry density after dipping, “$\rho_{H}$” is the total dry density after heat treatment, “$\Delta_{\rho_{1}-\rho_{H}}$” is the oven-dry density difference after heat treatment, “$K_{\rho_{1}-\rho_{H}}$” is after heat treatment is the coefficient of variation of the oven-dry density.

**Table 5:** Variance analysis of the total dry density of impregnated heat-treated wood.

Factor	Sum of Squares	Degree of Freedom	F Ratio	F Critical Value	*p*-Value	Significance
W	0.442	2	1	19	0.500	
H	11.949	2	27.034	19	0.036	**
T	0.762	2	1.724	19	0.367	
error	0.442	2				

Significant level α = 0.05.

**Table 6:** Visual analysis of oven-dry and air-dry shrinkage of impregnated heat-treated wood.

Number	W (%)	H (°C)	T (Lh)	Total Dry Shrinkage Rate (%)			Air Drying Shrinkage Rate (%)		
				Radial	Tangential Direction	Volume	Radial	Tangential Direction	Volume
1	0	160	2	6.3	6.4	12.5	3.6	3.8	7.3
2	0	180	4	5.2	5.3	10.2	2.7	2.8	5.4
3	0	200	6	4.1	4.7	8.7	1.8	2.0	3.8
4	1	160	4	6.5	7.3	13.3	3.9	4.3	8.0
5	1	180	6	4.3	5.5	9.9	2.2	2.9	5.0
6	1	200	2	4.8	5.7	10.3	2.6	3.1	5.6
7	2	160	6	6.0	6.2	12.2	3.6	3.7	7.3
8	2	180	2	5.4	6.5	11.7	3.0	3.6	6.8
9	2	200	4	4.5	4.8	9.3	2.2	2.4	4.5
Mean 1				5.2	6.267	5.5	2.7	3.7	3.067
Mean 2				5.2	4.967	5.4	2.9	2.633	2.933
Mean 3				5.3	4.467	4.8	2.933	2.2	2.533
RangeRadial				0.1	1.8	0.7	0.233	1.5	0.534
Mean 1				5.467	6.633	6.2	2.867	3.933	3.5
Mean 2				6.167	5.767	5.8	3.433	3.1	3.167
Mean 3				5.833	5.067	5.467	3.233	2.5	2.867
Range Rtangential				0.7	1.566	0.733	0.566	1.433	0.633
Mean 1				10.467	12.667	11.5	5.5	7.533	6.567
Mean 2				11.167	10.6	10.933	6.2	5.733	5.967
Mean 3				11.067	9.433	10.267	6.2	4.633	5.367
RangeRvolume				0.7	3.234	1.233	0.7	2.9	1.2

**Table 7:** Radial oven-dry shrinkage variance of impregnated heat-treated wood.

Factor	Sum of Squares	Degree of Freedom	F Ratio	F Critical Value	*p*-Value	Significance
W	0.02	2	1	19	0.500	
H	5.18	2	259	19	0.004	**
T	0.86	2	43	19	0.023	**
error	0.02	2				

Significant level α = 0.05.

**Table 8:** Tangential oven-dry shrinkage variance of impregnated heat-treated wood.

Factor	Sum of Squares	Degree of Freedom	F Ratio	F Critical Value	*p*-Value	Significance
W	0.736	2	1	9	0.500	
H	3.696	2	5.022	9	0.166	
T	0.809	2	1.099	9	0.476	
error	0.74	2				

Significant level α = 0.1.

**Table 9:** The variance of volume oven-dry shrinkage of impregnated heat-treated wood.

Factor	Sum of Squares	Degree of Freedom	F Ratio	F Critical Value	*p*-Value	Significance
W	0.86	2	1	9	0.500	
H	16.087	2	18.706	9	0.051	*
T	2.287	2	2.659	9	0.273	
error	0.86	2				

Significant level α = 0.1.

**Table 10:** Radial air drying shrinkage variance of impregnated heat-treated wood.

Factor	Sum of Squares	Degree of Freedom	F Ratio	F Critical Value	*p*-Value	Significance
W	0.096	2	1	9	0.500	
H	3.576	2	37.25	9	0.026	**
T	0.462	2	4.813	9	0.172	
error	0.1	2				

Significant level α = 0.1.

**Table 11:** Tangential air drying shrinkage variance of impregnated heat-treated wood.

Factor	Sum of Squares	Degree of Freedom	F Ratio	F Critical Value	*p*-Value	Significance
W	0.496	2	1	9	0.500	
H	3.109	2	6.268	9	0.138	
T	0.602	2	1.214	9	0.452	
error	0.5	2				

Significant level α = 0.1.

**Table 12:** Volume air drying shrinkage variance of impregnated heat-treated wood.

Factor	Sum of Squares	Degree of Freedom	F Ratio	F Critical Value	*p*-Value	Significance
W	0.98	2	1	9	0.500	
H	12.86	2	13.122	9	0.071	*
T	2.16	2	2.204	9	0.312	
error	0.98	2				

Significant level α = 0.1.

**Table 13:** Visual analysis of fully dry to the water-saturated swelling rate of impregnated heat-treated wood.

Number	W (%)	H (°C)	T (h)	Swelling Properties Rate (%)		
				Radial	Tangential Direction	Volume
1	0	160	2	4.0	4.2	7.7
2	0	180	4	4.0	4.0	7.7
3	0	200	6	3.3	3.9	7.3
4	1	160	4	4.0	4.9	9.2
5	1	180	6	3.3	4.4	7.7
6	1	200	2	3.4	4.3	7.5
7	2	160	6	4.2	4.3	8.7
8	2	180	2	4.0	5.0	8.7
9	2	200	4	3.4	3.9	7.5
Mean 1	3.767	4.067	3.8			
Mean 2	3.567	3.767	3.8			
Mean 3	3.867	3.367	3.6			
RangeRadial	0.3	0.7	0.2			
Mean 1	4.033	4.467	4.5			
Mean 2	4.533	4.467	4.267			
Mean 3	4.4	4.033	4.2			
RangeRTangential	0.5	0.434	0.3			
Mean 1	7.567	8.533	7.967			
Mean 2	8.133	8.033	8.133			
Mean 3	8.3	7.433	7.9			
RangeRvolume	0.733	1.1	0.233			

**Table 14:** The variance of radial swelling property of impregnated heat-treated wood.

Factor	Sum of Squares	Degree of Freedom	F Ratio	F Critical Value	*p*-Value	Significance
W	0.14	2	1.75	9	0.364	
H	0.74	2	9.25	9	0.098	*
T	0.08	2	1	9	0.500	
error	0.08	2				

Significant level α = 0.1.

**Table 15:** The variance of the tangential swelling property of impregnated heat-treated wood.

Factor	Sum of Squares	Degree of Freedom	F Ratio	F Critical Value	*p*-Value	Significance
W	0.402	2	2.698	9	0.270	
H	0.376	2	2.523	9	0.284	
T	0.149	2	1	9	0.500	
error	0.15	2				

Significant level α = 0.1.

**Table 16:** The variance of volume swelling property of impregnated heat-treated wood.

Factor	Sum of Squares	Degree of Freedom	F Ratio	F Critical Value	*p*-Value	Significance
W	0.887	2	10.195	9	0.089	*
H	1.82	2	20.92	9	0.046	**
T	0.087	2	1	9	0.500	
error	0.09	2				

Significant level α = 0.1.

**Table 17:** Orthogonal analysis of MOR and MOE of impregnated heat-treated wood.

Number	W (%)	H (°C)	T (h)	ρ (g/cm^3^)	ω	MOR (MPa)		MOE (MPa)	
						ω	12%	ω	12%
1	0	160	2	0.592	11.3	89.6	87.1	10122	10018
2	0	180	4	0.622	9.2	96.5	85.6	9493	9092
3	0	200	6	0.590	8.6	77.4	67.0	8272	7857
4	1	160	4	0.548	11.2	92.8	89.8	9762	9645
5	1	180	6	0.601	9	88.3	77.8	9154	8746
6	1	200	2	0.586	8.9	91.1	80.1	9564	9131
7	2	160	6	0.571	9.1	98.4	87.2	9949	9521
8	2	180	2	0.566	9.4	77.7	69.7	8703	8368
9	2	200	4	0.534	9.1	80.5	71.0	8299	7933
Mean 1	79.9	88.033	78.967						
Mean 2	82.567	77.7	82.133						
Mean 3	75.967	72.7	77.333						
RMOR	6.6	15.333	4.8						
Mean 1	8989	9728	9172.33						
Mean 2	9174	8735.33	8890						
Mean 3	8607.33	8307	8708						
RMOE	566.667	1421	464.333						

**Table 18:** The variance of MOR of impregnated heat-treated wood.

Factor	Sum of Squares	Degree of Freedom	F Ratio	F Critical Value	*p*-Value	Significance
W	66.142	2	1.851	9	0.351	
H	366.889	2	10.267	9	0.089	*
T	35.736	2	1	9	0.500	
error	35.74	2				

Significant level α = 0.1.

**Table 19:** The variance of MOE of impregnated heat-treated wood.

Factor	Sum of Squares	Degree of Freedom	F Ratio	F Critical Value	*p*-Value	Significance
W	501005.556	2	1.525	9	0.396	
H	3188097.556	2	9.707	9	0.093	*
T	328441.556	2	1	9	0.500	
error	328441.56	2				

Significant level α = 0.1.

## Data Availability

The data presented in this study are available on request from the corresponding author.
